# Asthma manifestations, health-related factors, and sickness absence: A national register-based study

**DOI:** 10.1016/j.jacig.2026.100774

**Published:** 2026-08-10

**Authors:** Sandra Ekström, Maria Ödling, Gun Johansson, Jon R. Konradsen, Caroline Stridsman, Inger Kull

**Affiliations:** a Department of Clinical Science and Education, Karolinska Institutet, Södersjukhuset, Stockholm, Sweden; b Center for Occupational and Environmental Medicine, Region Stockholm, Stockholm, Sweden; c Institute of Environmental Medicine, Karolinska Institutet, Stockholm, Sweden; d Department of Women’s and Children’s Health, Karolinska Institutet, Stockholm, Sweden; e Pediatric Allergy and Pulmonology Unit at Astrid Lindgren Children’s Hospital, Karolinska University Hospital, Stockholm, Sweden; f Department of Public Health and Clinical Medicine, The OLIN and Sunderby Research Unit, Umeå University, Umeå, Sweden; g Sachs’ Children and Youth Hospital, Södersjukhuset, Stockholm, Sweden

**Keywords:** Asthma control, health-related factors, lung function, sickness absence

## Abstract

**Background:**

There is limited evidence on how asthma manifestations and health-related factors influence sickness absence among patients with asthma.

**Objective:**

We sought to investigate the impact of asthma control, lung function, allergy, and health-related factors (overweight/obesity, physical activity, and smoking) on sickness absence among adult patients with asthma. A secondary aim was to estimate productivity losses related to sickness absence for uncontrolled asthma.

**Methods:**

The study included 69,548 patients with asthma 18 to 65 years old registered in the Swedish National Airway Register during 2021-2022. Data on asthma manifestations and health-related factors were obtained from the most recent health care visit. Sickness absence (>14 days) during 2022 was obtained from Statistics Sweden. Analyses were performed with logistic regression models using multiple imputations. Productivity losses were calculated using the human capital approach.

**Results:**

Overall, 17.4% of patients had any sickness absence. Uncontrolled asthma was associated with increased odds of sickness absence compared with controlled asthma (adjusted odds ratio, 1.46; 95% CI, 1.37-1.56), whereas no association was observed for low lung function or allergy. Overweight, obesity, low physical activity, and smoking were independently associated with sickness absence. Individuals with uncontrolled asthma had more days of sickness absence (19.3 vs 11.2, *P* < .001) and excess productivity losses (estimated at €144 per individual after adjustment for age and sex) compared with individuals with controlled asthma.

**Conclusions:**

Uncontrolled asthma and adverse health-related factors were independently associated with sickness absence and related productivity losses among working-age patients with asthma. Improving asthma control and modifiable health-related factors may reduce sickness absence and societal costs.

Asthma affects approximately 10% of the adult population in Sweden and accounts for substantial morbidity and productivity losses worldwide.^1^^,^^2^ The goals of asthma management are to achieve symptom control and minimize the risk of long-term negative consequences of the disease.^3^ However, a recent study indicates that more than 50% of patients treated for mild to moderate asthma have uncontrolled disease,^4^ which contributes to limitations in daily activities, impaired sleep, and reduced quality of life.^5^

A high burden of asthma symptoms and low disease control may also negatively affect several aspects of working life.^6^, ^7^, ^8^, ^9^, ^10^ Recent results from the population-based BAMSE (Barn/Children Allergi/Allergy Milieu Stockholm Epidemiologic) study cohort showed that asthma was associated with higher sickness absence (periods >14 days) and productivity losses among young adults. Sickness absence was particularly high among individuals with uncontrolled or persistent asthma and those with concurrent rhinitis.^11^ Based on the same cohort, asthma was also associated with increased risk of short-term sickness absence and presenteeism (defined as working despite being unwell).^12^ However, population-based studies included relatively few participants and only young adults, which limited power and the generalizability of the findings. As the impact of asthma on working life may differ by factors such as age, sex, and socioeconomic status, there is a need for larger studies that include the whole range of the working population.

Moreover, less is known about factors that could reduce or exacerbate the negative consequences of asthma in working life. Other characteristics beyond asthma control such as lung function and allergic comorbidity may also influence work-related outcomes. Reduced lung function is associated with more severe symptoms and exacerbations, whereas concomitant allergy and rhinitis are common in asthma and may contribute to additional symptom burden.^13^ A potential influence of asthma on working life may also be explained by health-related factors such as physical activity, smoking status, and obesity, which may affect both asthma severity and general work ability.^14^ In the BAMSE study, sickness absence was most common in the group with both asthma and obesity, whereas no difference was observed related to physical activity.^11^ However, a Norwegian cross-sectional study observed that asthma and obesity were independently associated with symptom scores and lung function, whereas working ability scores were negatively associated only with asthma, not with body mass index (BMI).^15^ Thus, the role of obesity and other health-related factors in the association between asthma and work-related outcomes including sickness absence requires further investigation.

To address these knowledge gaps, register-based studies offer advantages as they cover large, unselected populations including objective data on asthma and sickness absence. The aim of the present study was to investigate how asthma control, lung function, and allergy as well as health-related factors (BMI, physical activity, and smoking status) affect sickness absence among patients with asthma registered in the Swedish National Airway Register (SNAR).^16^ A secondary aim was to estimate the productivity losses related to sickness absence and population attributable fraction (PAF) for uncontrolled asthma.

## Methods

### Study design and study population

This cross-sectional study was based on data from SNAR, a national quality register established in 2013. SNAR includes data on more than 500,000 pediatric and adult patients diagnosed with asthma (*International Classification of Diseases, Tenth Revision* code J45) or chronic obstructive lung disease (COPD). Although registration is not mandatory and coverage is not complete, the register includes data from more than 1,000 clinics across Sweden (primary, secondary, or tertiary care) and is considered broadly representative of routine clinical care for asthma and COPD in Sweden.^16^^,^^17^ Registrations in SNAR include information on background characteristics as well as data from clinical visits such as Asthma Control Test (ACT), allergy, lung function, BMI, smoking status, and physical activity level. In addition, SNAR is linked to the Longitudinal Integrated Database for Health Insurance and Labour market studies from Statistics Sweden for information on sickness absence (from the Swedish Social Insurance Agency), highest completed education (from the Swedish Register of Education), and socioeconomic group based on occupation (from the the Swedish Occupational Register).^18^

The study population comprised 69,548 patients 18 to 65 years old (workers and nonworkers) with a recorded asthma diagnosis and at least 1 visit to a primary care provider or specialized outpatient care provider between January 1, 2021, and December 31, 2022. For individuals with multiple visits during this period, data from the most recent visit were obtained. Patients with a recorded diagnosis of COPD at any time were excluded. The study was approved by the Swedish Ethical Review Authority (Dnr 2019-04915, Dnr 2020-00508).

### Definition of exposure variables from SNAR (asthma control, lung function, allergy, and health-related factors)

Asthma control was assessed using the ACT, which ranges from 5 to 25 points with <20 points indicating uncontrolled asthma.^19^ Low lung function was defined as FEV_1_ <80% of predicted values based on spirometry measurements.^20^^,^^21^ Allergy was defined as having a recorded allergy in SNAR, as registered by health care professionals at the clinical visit. BMI was categorized in accordance with the World Health Organization definitions (underweight: <18.5 kg/m^2^; normal weight: 18.5-24.9 kg/m^2^; overweight: 25-29.9 kg/m^2^; obesity: ≥30 kg/m^2^). The underweight category was combined with normal weight due to the low number of underweight individuals. Physical activity was based on the recorded number of days per week with at least 30 minutes of physical activity (categorized as 0-1 day, 2-6 days, and 7 days). Smoking status was categorized as never, past, occasional, or daily smoker.

### Definition of outcome variables from Longitudinal Integrated Database for Health Insurance and Labour Market Studies (sickness absence and related productivity losses)

Sickness absence was defined as a period of consecutive benefit payments longer than 14 days for sickness absence, preventive rehabilitation, or occupational injury in 2022. Productivity losses related to sickness absence in 2022 were estimated from a societal perspective,^22^ reflecting the costs to society of the hypothetical lost production during sickness absence from work. Productivity losses of society were estimated using the human capital approach based on the average cost of employment.^22^^,^^23^ This was calculated by multiplying the number of net days with sickness absence (where net days represent full-day equivalents, with part-time sickness absence weighted according to compensation level), converted into months, among individuals classified as workers by the sum of the average monthly salary in 2022 (€3,602 calculated using the 2022 exchange rate: €1 = kr10.6317)^24^ and the social security contribution from employers (31.42%).^25^

### Definition of covariates

Age was categorized into 3 groups: 18 to 29 years, 30 to 44 years, and 45 to 65 years. Education level was categorized as elementary school, upper secondary school, and university. Socioeconomic group was derived from European socioeconomic classification, which is based on registered occupation. We categorized the variable following the recommendation of Statistics Sweden as high class (groups 1 and 2), middle class (groups 3 and 4), and working class (groups 5, 6, and 7).^18^ As nonworkers are not included in this variable, individuals with missing data were included in a separate category labeled nonworkers. Comorbidity was defined as having a recorded diagnosis such as diabetes, heart disease, hypertension, osteoporosis, and sleep apnea in SNAR.

### Statistical analyses

Descriptive statistics are presented as numbers and percentages. Differences in background variables by sickness absence status were tested using the χ^2^ test. Associations between asthma phenotypes and health-related factors with any period of sickness absence >14 days (yes/no) were analyzed by logistic regression models, expressed as odds ratio (OR) with 95% CI. Two models were fitted: a crude model and an adjusted model including age, sex, education, socioeconomic group, BMI status, physical activity, smoking status, and comorbidity. The statistical method Multiple Imputation by Chained Equations^26^ was used to fill in missing data, with 100 datasets generated. Frequency of missing data and differences between imputed and nonimputed variables are shown in Table E1 (in this article’s online Repository at www.jaci-global.org). Stratified analyses in relation to uncontrolled asthma were performed for sex, age group, and socioeconomic group. Interaction terms between these variables and asthma control were included to assess effect modification, and joint significance of the interaction terms was tested by Wald tests. As a sensitivity analysis, we restricted the analyses to participants who were workers, as patterns of sickness absence may differ between individuals who are working and individuals who are unemployed, studying, or otherwise outside the workforce.

The PAF for sickness absence >14 days related to uncontrolled asthma was estimated to quantify the proportion of sickness absence that could theoretically be avoided if asthma were controlled, assuming a causal relationship. PAF was calculated using the formula $\text{PAF}=\frac{P_{e}\;\left(\text{RR}-1\right)}{P_{e}\;\left(\text{RR}-1\right)+1}$, where $P_{e}$ represents the prevalence of uncontrolled asthma in the study population. Adjusted ORs from multivariable logistic regression were used as approximations of relative risks.

Average estimated costs related to sickness absence >14 days among individuals with uncontrolled and controlled asthma were reported together with bias-corrected bootstrap 95% CIs based on 2,000 iterations.^27^ Because the cost data were non-normally distributed, differences between groups were assessed using Wilcoxon rank sum test, a nonparametric method appropriate for skewed distributions. Excess net days of sickness absence and excess productivity losses associated with asthma were estimated using zero-inflated Poisson regression, with adjustment for age and sex.^28^ Productivity losses were estimated only among individuals classified as workers, as the cost calculations were based on salary. All analyses were performed with Stata version 16.1 (StataCorp LLC, College Station, Texas). *P* values <.05 were considered statistically significant.

## Results

### Study population

Of the study population (N = 69,548), 61% were women with a mean (SD) age of 44.5 (13.6) years. More than half (54.7%) of the study population had a university education, and 80.6% were workers. Approximately 2 out of 3 (68.2%) patients were overweight or obese, and 9.8% were daily smokers. The majority of patients (98.4%) were registered in primary care. Almost half (43.7%) had uncontrolled asthma, and a similar proportion had low lung function (42.7%) or allergy (41.7%). One in 4 (25.5%) had comorbidities such as diabetes, heart disease, hypertension, osteoporosis, and sleep apnea. Characteristics of the study population are displayed in Table I.

**Table I:** Characteristics of study population in relation to sickness absence (N = 69,548)

Characteristics	No sickness absence (n = 57,436)∗	Sickness absence (n = 12,112)∗	Total (N = 69,548)∗	*P* value†
Female sex, no. (%)	33,640 (58.6)	8,756 (72.3)	42,396 (61.0)	<.001
Age group, no. (%)				<.001
18-29 y	11,541 (20.1)	1,261 (10.4)	12,802 (18.4)	
30-44 y	15,128 (26.3)	3,545 (29.3)	18,673 (26.9)	
45-65 y	30,767 (53.6)	7,306 (60.3)	38,073 (54.7)	
Education level, no. (%)				<.001
Elementary school	6,939 (12.2)	1,324 (11.0)	8,263 (18.4)	
Upper secondary school	24,680 (43.5)	5,883 (48.7)	30,563 (44.4)	
University	25,167 (44.3)	4,866 (40.3)	30,033 (43.6)	
Socioeconomic group, no. (%)‡				<.001
High class	16,108 (28.1)	3,072 (25.4)	19,180 (27.6)	
Middle class	7,504 (13.1)	1,475 (12.2)	8,979 (12.9)	
Working class	21,651 (37.7)	6,279 (51.8)	27,930 (40.2)	
Nonworking	12,173 (21.2)	1,286 (10.6)	13,459 (19.4)	
BMI category, no. (%)				<.001
Normal weight/underweight	11,811 (33.3)	1,824 (24.5)	13,635 (31.8)	
Overweight	12,061 (34.0)	2,397 (32.2)	14,458 (33.7)	
Obese	11,563 (32.6)	3,217 (43.3)	14,780 (34.5)	
Physical activity (days per week with ≥30 min), no. (%)				<.001
0-1 d	5,378 (19.5)	1,409 (25.0)	6,787 (20.5)	
2-6 d	11,165 (40.5)	2,075 (36.9)	13,240 (39.9)	
7 d	11,010 (40.0)	2,143 (38.1)	12,153 (39.6)	
Smoking status, no. (%)				<.001
Never	26,483 (66.0)	4,907 (59.3)	31,390 (64.9)	
Past	9,006 (22.5)	2,127 (25.7)	11,133 (23.0)	
Occasionally	926 (2.3)	196 (2.4)	1,122 (2.3)	
Daily	3,708 (9.2)	1,041 (12.6)	4,749 (9.8)	
Level of care, no. (%)				.10
Primary care	56,519 (98.4)	11,944 (98.6)	68,463 (98.4)	
Specialized outpatient care	917 (1.6)	168 (1.4)	1,085 (1.6)	
Asthma control, no. (%)				<.001
Controlled asthma (ACT ≥20)	15,578 (58.3)	2,410 (46.4)	17,988 (56.3)	
Uncontrolled (ACT ≤19)	11,164 (41.8)	2,786 (53.6)	13,950 (43.7)	
FEV_1_ pre <80% of predicted, no. (%)	11,183 (42.3)	2,329 (44.8)	13,512 (42.7)	.001
Allergy diagnosis, no. (%)	24,015 (41.8)	4,965 (41.0)	28,980 (41.7)	.10
Comorbidity, no. (%)§	14,013 (24.4)	3,736 (30.9)	17,749 (25.5)	<.001

*FEV*_*1*_*pre,* Pre-bronchodilator FEV_1_.

∗ Some numbers may not add up to total due to missing data.

† *P* values obtained by χ^2^ test.

‡ Socioeconomic group based on occupation.

§ Comorbidities include diabetes, heart disease, hypertension, osteoporosis, and sleep apnea.

In total, 17.4% of the study population experienced any period of sickness absence >14 days (20.7% in women, 12.4% in men). Among patients with sickness absence, the median duration was 49 days. Compared with individuals without sickness absence, those with sickness absence were older, were more often female, had lower level of education, were more often obese, and had lower levels of physical activity. Individuals with sickness absence were also more likely to have uncontrolled asthma, low lung function, and comorbidities (diabetes, heart disease, hypertension, osteoporosis, and sleep apnea), although there was no difference in the proportion of allergy (Table I).

### Associations between asthma manifestations and sickness absence

**Fig 1:**
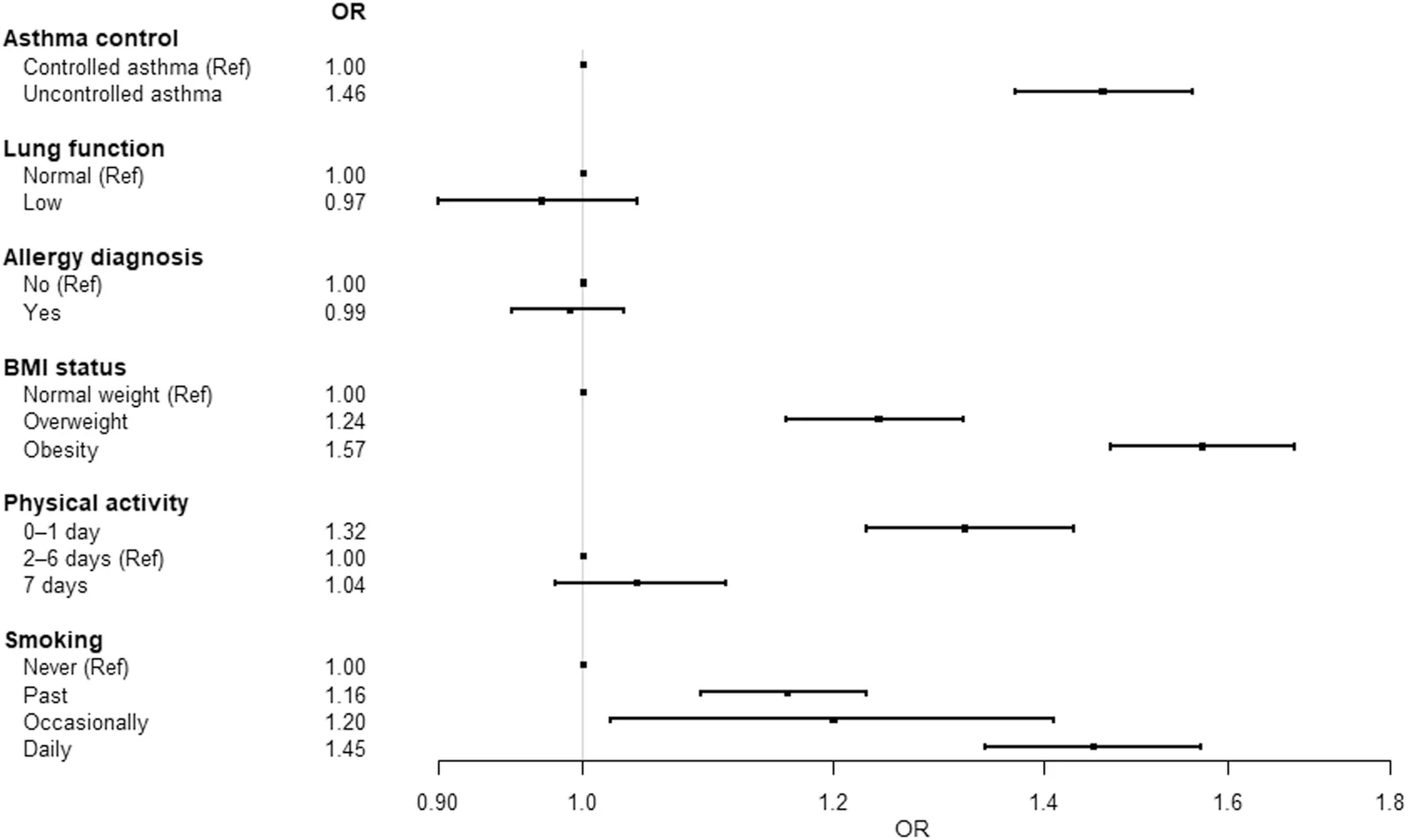
Associations between asthma phenotypes and health-related factors in relation to sickness absence (N = 69,548). Adjusted for sex, age, education, socioeconomic group, BMI status, physical activity, smoking status, and comorbidity (diabetes, heart disease, hypertension, osteoporosis, and sleep apnea). Missing data were handled using Multiple Imputation by Chained Equations, with 100 datasets generated. Points represent ORs, and horizontal lines represent 95CIs. *Ref,* Reference.

In both crude and adjusted analyses, uncontrolled asthma was associated with higher odds of sickness absence (adjusted OR, 1.46; 95% CI, 1.37-1.56) compared with controlled asthma, whereas no association was observed for allergy (Fig 1; Table E2 in the Online Repository at www.jaci-global.org). In crude analyses, there was a positive association between sickness absence and low lung function; however, this association was fully attenuated in the adjusted model (adjusted OR, 0.97; 95% CI, 0.90-1.04). The complete-case analysis without imputed data (n = 19,761) yielded similar results (eg, adjusted OR, 1.47; 95% CI, 1.36-1.59 for uncontrolled asthma). Results were also similar when the analyses were restricted to participants who were employed (Table E2). Interaction analyses indicated a somewhat stronger association between uncontrolled asthma and sickness absence among men (adjusted OR, 1.55; 95% CI, 1.39-1.72) compared with women (adjusted OR, 1.42; 95% CI, 1.32-1.52) (*P* value for interaction = .009), although CIs partly overlapped. In contrast, no significant difference was observed in relation to age or socioeconomic group (Table II).

**Table II:** Associations between asthma control (uncontrolled vs controlled asthma) and sickness absence stratified by sex, age group, and socioeconomic group based on occupation

	OR (95% CI)∗	*P*_int_∗
Sex		.009
Female (n = 42,396)	1.42 (1.33-1.52)	
Male (n = 27,152)	1.55 (1.39-1.72)	
Age group		.54
18-29 y (n = 12,802)	1.42 (1.21-1.68)	
30-44 y (n = 18,673)	1.45 (1.31-1.59)	
45-65 y (n = 38,073)	1.45 (1.35-1.57)	
Socioeconomic group†		.75
High class (n = 19,180)	1.36 (1.23-1.52)	
Middle class (n = 8,979)	1.44 (1.24-1.68)	
Working class (n = 27,930)	1.48 (1.37-1.60)	
Nonworkers (n = 13,459)	1.66 (1.42-1.94)	

P_*int*_*, P* value for interaction.

∗ *P* value for interaction obtained by Wald test.

† Based on occupation.

### Associations between health-related factors and sickness absence

High BMI status (overweight and obesity), low physical activity, and smoking status (past, occasional, and daily) all were associated with having any sickness absence longer than 14 days (Fig 1; Table E2). The adjusted OR for obesity compared with normal weight/underweight was 1.57 (95% CI, 1.47-1.68), and the corresponding OR for daily smoking compared with never smoking was 1.45 (95% CI, 1.34-1.57) in the fully adjusted models. Similar results were observed in the complete-case analysis and when restricting the analyses to workers only (Table E2).

### Estimated cost of sickness absence in relation to asthma control

Table III presents estimates of productivity losses related to net days of sickness absence according to asthma control among individuals classified as workers. The mean number of net days of sickness absence was higher among individuals with uncontrolled asthma (19.3 days) than among individuals with controlled asthma (11.2 days). Correspondingly, estimated productivity losses were significantly greater in the uncontrolled asthma group (€3,011 per person) compared with the controlled asthma group (€1,742 per person). Table IV presents excess productivity costs estimated using zero-inflated Poisson regression. After adjustment for age and sex, the estimated average excess productivity loss was €144 per person (95% CI, 105-189) among individuals with uncontrolled asthma compared with individuals with controlled asthma.

**Table III:** Net days with sickness absence in 2022 and estimated costs in euros among participants classified as workers

Asthma control	Net days with sickness absence		Cost of sickness absence (€)		*P* value†
	Mean	95% CI∗	Mean	95% CI∗	
Controlled asthma (n = 11,083)	11.2	10.5-11.9	1742.2	1633.2-1861.2	<.001
Uncontrolled asthma (n = 15,387)	19.3	18.2-20.4	3011.2	2834.6-3182.4	
Overall (n = 56,089)	16.5	16.0-16.9	2565.1	2491.1-2635.9	

∗ Bias-corrected bootstrap CIs, 2000 iterations.

† *P* value for difference in costs obtained through Wilcoxon rank-sum tests.

**Table IV:** Excess net days with sickness absence in 2022 and estimated excess costs related to uncontrolled compared with controlled asthma among participants classified as workers in euros estimated through zero-inflated Poisson regression

	Crude model (n = 26,470)		Adjusted for age and sex (n = 26,470)	
	β	95% CI	β	95% CI
Excess net days with sickness absence in 2022	8.2	6.8-9.5	0.92	0.67-1.21
Excess cost of sickness absence in 2020 and 2021 (€)	1269.0	1053.2-1476.5	143.7	104.9-188.6

### PAF of uncontrolled asthma

Based on the prevalence of uncontrolled asthma (43.7%) and the adjusted OR for sickness absence (1.46), the PAF was estimated at approximately 17%. Assuming a causal relationship, this suggests that about 17% of all sickness absence among adults with asthma could potentially be prevented if patients with uncontrolled asthma achieved asthma control. From an economic perspective, the mean annual productivity loss per worker was €2,561, corresponding to a total annual cost of approximately €144 million among the 56,089 workers in this population. Applying the PAF, a 17% reduction in sickness absence would translate into a potential cost saving of approximately €24 million. In contrast, using estimates from the zero-inflated Poisson regression, which accounted for differences in age and sex, the estimated annual excess productivity cost attributable to uncontrolled asthma among workers in this population was lower, at approximately €3.4 million (excess cost €144 per person among an estimated 23,900 workers with uncontrolled asthma).

## Discussion

In this national register–based study of working-age adults with asthma, we found that uncontrolled disease was strongly associated with sickness absence >14 days. In addition, several health-related factors including overweight/obesity, smoking status, and low physical activity were independently associated with sickness absence. In contrast, no clear associations were observed for reduced lung function or allergy. Taken together, these findings suggest that symptom control and modifiable health-related factors may play an important role in reducing sickness absence and related societal costs among adults with asthma.

Our results are consistent with previous studies showing that poor asthma control is a key determinant of sickness absence and impaired work ability.^7^^,^^9^^,^^10^ For example, a large Danish register-based study of patients with asthma demonstrated that uncontrolled asthma (defined by treatment and exacerbations) was associated with temporary sick leave, unemployment, and disability benefits.^7^ Similarly, a small Dutch study of patients with asthma showed that psychosocial factors and asthma symptoms was associated with sick leave, whereas lung function measures were not associated with sick leave or change in sick leave over time.^29^^,^^30^ One explanation may be that asthma control captures patient-reported symptoms, which may be more directly related to activity limitations and work capacity than spirometry measures, which primarily reflects physiologic impairment at a single time point.

The productivity costs in this population of patients with asthma were estimated at €2,565 per person. This can be compared with estimates for costs related to absenteeism reported in Spain (€286/month)^31^ and the United Kingdom (£1,012 for well-controlled asthma and £2,747 for poorly controlled asthma).^32^ Uncontrolled asthma was associated with excess productivity losses compared with controlled asthma, which has also been observed in other studies.^32^ However, this association was attenuated after adjustment for age and sex with estimated annual excess costs at €144 per person. This may be explained by sex difference in asthma control and sickness absence, as women in our study had both poorer asthma control and more days of sickness absence.

In the total working population of Sweden, it can be estimated that approximately 250,000 individuals have uncontrolled asthma (assuming a prevalence of 5% [50% among 10% with asthma]). This would translate into a total excess annual productivity cost of approximately €36 million, which can be compared with the total cost of asthma, which has been estimated to be approximately €660 million (kr7 billion).^33^ From a population perspective, our estimates also suggest that approximately one-sixth of sickness absence among working-age adults with asthma may be attributable to uncontrolled disease, although this estimate should be interpreted cautiously given the cross-sectional design.

Previous studies have indicated that there may be a stronger association between asthma and sick leave in women compared with men.^6^^,^^9^ However, whether there is a sex-related difference in sick leave related to asthma control is unclear. In the present study, although the absolute prevalence of sickness absence was higher among women, the association between uncontrolled asthma and sickness absence appeared to be slightly stronger among men. Although this difference was modest, it may reflect sex-related differences in job types, work demands, or occupational exposures.

The lack of association for allergy is in line with a Finnish study showing that the impact of asthma and allergic rhinitis combined on the risk of sick leave days was marginal compared with asthma alone.^34^ However, in a previous study of young adults, stronger associations were found between allergic asthma compared with nonallergic asthma as well as asthma combined with rhinitis compared with asthma alone.^11^^,^^12^ The difference may be explained by the broad way in which allergies are recorded in the register, which includes heterogeneous allergic conditions such as food and drug allergies that may have limited relevance for work ability.

The strong associations between health-related factors and sickness absence should be highlighted. Obesity, in particular, has been linked to impaired work ability in the general population,^35^ but evidence among patients with asthma has been limited and mixed. A cross-sectional study of 626 patients with asthma in Norway found no association between obesity and sick leave or work ability.^15^ However, another Norwegian study found that the association between lifestyle risk score (including diet, physical activity, BMI, and smoking status) and sick leave was stronger among individuals with asthma compared with individuals without asthma,^14^ concluding that lifestyle changes may be particularly important for employees with asthma.

The major strengths of this study include the large sample size (N = 69,548) from a nationwide register including objective clinical data such as lung function and ACT, as well as linkage to detailed socioeconomic information. However, as registration in SNAR is voluntary and coverage is incomplete, selection bias cannot be excluded and should be considered when interpreting the findings. Nevertheless, the nationwide inclusion of patients from both primary and secondary care supports the relevance of the results to routine clinical practice, and the broad representation of patients supports generalizability.

Several limitations should be considered. The absence of a control group without asthma prevents direct estimation of the excess risk of sickness absence attributable to asthma. In addition, the cross-sectional design limits causal inference and prevents determination of the temporal sequence between exposures and outcomes. Consequently, reverse causation cannot be ruled out, and bidirectional relationships may exist between asthma control, health-related factors, and sickness absence. For example, poor asthma control may reduce physical activity, and low physical activity may also contribute to poorer asthma outcomes. Furthermore, sickness absence periods shorter than 14 days were not available, which may be differently associated with asthma manifestations and health-related factors. The sickness absence data were also not diagnosis specific, and therefore we could not determine whether absence was directly attributable to asthma or to other health conditions. Consequently, the observed associations may partly reflect the overall health burden associated with uncontrolled asthma and adverse health-related factors rather than asthma-specific mechanisms alone. We also lacked information on workplace characteristics and occupational exposures, including the possibility for distance work, which may influence work ability and risk of sickness absence. A further limitation is that asthma control was defined solely using ACT scores. Although ACT is a validated measure of symptom control, it does not account for exacerbations or treatment intensity and may therefore not fully reflect overall asthma control. In addition, the allergy variable reflects the presence of any recorded allergy and does not provide information on allergy type, sensitization profile, or severity. Finally, several variables contained a substantial amount of missing data. Missing data were handled using multiple imputation, which assumes that data are missing at random. This assumption may not have been fully met for some variables; however, imputed and complete-case analyses showed similar results indicating that missing data were unlikely to have introduced substantial bias.

### Conclusions

Uncontrolled asthma and adverse health-related factors were associated with an increased risk of sickness absence among adults with asthma. Uncontrolled asthma may also be associated with higher productivity losses related to sickness absence. Approximately one-sixth of all sickness absence was estimated to be attributable to uncontrolled asthma. These findings suggest that improving asthma control and addressing modifiable health-related factors may have the potential to reduce sickness absence among adults with asthma. Future longitudinal studies are needed to clarify the direction of these associations and determine whether improvements in asthma control and health-related behaviors lead to reduced sickness absence. This information may inform targeted preventive strategies in health care and occupational settings.

### Declaration of generative AI and AI-assisted technologies

During the preparation of this work, the authors used Microsoft Copilot to improve the language and clarity of the manuscript. After using this tool, the authors reviewed and edited the content as needed and take full responsibility for the content of the published article.

## Disclosure statement

This study was supported by grants from the Swedish Asthma and Allergy Research Foundation (F2024-0020). C.S. was supported by the 10.13039/501100003793Swedish Heart-Lung Foundation (20230537, 20230473), Region Norrbotten, and through a regional agreement between Region Västerbotten and Umeå University (ALF).

Disclosure of potential conflict of interest: J.R. Konradsen reports advisory board fees from Novartis and ALK and institutional fees from Regeneron Pharmaceuticals and Thermo Fisher Scientific outside the submitted work. The rest of the authors declare that they have no relevant conflicts of interest.

## Supplementary data

Supplementary Table E1

Supplementary Table E2
