# Supplementary material for: Asthma manifestations, health-related factors, and sickness absence: A national register-based study
Source: J Allergy Clin Immunol Glob. 2026 Aug 10;5(6):100774. doi: 10.1016/j.jacig.2026.100774 (PMC13528352; doi:10.1016/j.jacig.2026.100774)
Supplement: Supplementary file 1 [file mmc1.docx]

**Table E1**. Characteristics of the study population (N = 69,548): complete-case and multiply imputed data

|  | **Non-imputed** | **Imputed** |
| --- | --- | --- |
| *Characteristics* | **n (%)** | **%^1^** |
| **Sex**   - Female   *Missing* | 42,396 (61.0)  0 | - |
|  |  |  |
|  |  |  |
| **Age group**   - 18-29 years - 30-44 years - 45-65 years   *Missing* | 12,802 (18.4)  18,673 (26.9)  38,073 (54.7)  0 | - |
| **Education level**   - Elementary school - Upper secondary school - University   *Missing* | 8,263 (12.0)  30,563 (44.4)  30,033 (43.6)  689 | 12.1  44.4  43.5  0 |
| **Socioeconomic group based on occupation**   - High class - Middle class - Working class - Non-working   *Missing* | 19,180 (27.6)  8,979 (27.6)  27,930 (40.2)  13,459 (19.4)  0 | - |
| **BMI category**   - Normal weight - Overweight - Obese   *Missing* | 13,635 (31.8)  14,458 (33.7)  14,780 (34.5)  26,675 | 33.4  33.7  32.8  0 |
| **Physical activity** (days per week with ≥30 min)   - 0-1 day - 2-6 days - 7 days   *Missing* | 6,787 (20.5)  13,240 (39.9)  13,153 (39.6)  36,368 | 20.4  40.0  39.7  0 |
| **Smoking**   - Never - Past - Occasionally - Daily   *Missing* | 31,390 (64.9)  11,133 (23.0)  1,122 (2.3)  4,749 (9.8)  21,154 | 65.3  22.7  2.4  9.7  0 |
| **Level of care**   - Primary care - Specialized outpatient care   *Missing* | 68,463 (98.4)  1,085 (1.6)  0 | - |
| **Asthma control**   - Controlled asthma (ACT≥20) - Uncontrolled (ACT<20)   *Missing* | 17,988 (56.3)  13,950 (43.7)  37610 | 55.4  44.6  0 |
| **FEV_1_ pre <80% of predicted** (n=31,649)  **Missing** | 13,512 (42.7)  37,899 | 43.3  0 |
| **Allergy diagnosis**  *Missing* | 28,980 (41.7)  0 | - |
| **Comorbidity^2^**  *Missing* | 17,749 (25.5)  0 | - |

1. Percentages are based on pooled estimates from multiply imputed data
2. Diabetes, heart disease, hypertension, osteoporosis, sleep apnoea
