# Supplementary material for: Asthma manifestations, health-related factors, and sickness absence: A national register-based study
Source: J Allergy Clin Immunol Glob. 2026 Aug 10;5(6):100774. doi: 10.1016/j.jacig.2026.100774 (PMC13528352; doi:10.1016/j.jacig.2026.100774)
Supplement: Supplementary file 2 [file mmc2.docx]

**Table E2.** Associations between asthma manifestations and health-related factors in relation to any sickness absence (N=69,548) in complete-case and multiply imputed data

|  | **Crude**  (N=69,548) | **Fully adjusted^1^**  (N=69,548) | **Fully adjusted workers only** (n=56,089) | **Fully adjusted^1^**  **Complete case analysis**  (n=19,761) |
| --- | --- | --- | --- | --- |
|  | **OR (95% CI)** | **OR (95% CI)** | **OR (95% CI)** | **OR (95% CI)** |
| **Asthma control**   - Controlled asthma - Uncontrolled asthma | Ref  **1.60 (1.50-1.70)** | Ref  **1.46 (1.37-1.55)** | Ref  **1.44 (1.35-1.53)** | Ref  **1.47 (1.36-1.59)** |
| **Lung function**   - Normal (FEV_1_ ≥80 pp) - Low (FEV_1_ <80 pp) | Ref  **1.10 (1.03-1.17)** | Ref  0.97 (0.90-1.04) | Ref  0.97 (0.91-1.05) | Ref  0.96 (0.89-1.03) |
| **Allergy diagnosis**   - No - Yes | Ref  0.97 (0.93-1.01) | Ref  0.99 (0.95-1.03) | Ref  1.03 (0.99-1.08) | Ref  1.01 (0.95-1.08) |
| **BMI status**   - Normal weight/underweight - Overweight - Obesity | Ref  **1.28 (1.20-1.36)**  **1.80 (1.69-1.91)** | Ref  **1.24 (1.16-1.32)**  **1.57 (1.47-1.68)** | Ref  **1.23 (1.14-1.31)**  **1.57 (1.46-1.69)** | Ref  **1.23 (1.13-1.34)**  **1.57 (1.44-1.71)** |
| **Physical activity**   - 0-1 day - 2-6 days - 7 days | **1.41 (1.31-1.53)**  Ref  1.05 (0.99-1.12) | **1.32 (1.23-1.43)**  Ref  1.04 (0.98-1.11) | **1.32 (1.22-1.43)**  Ref  1.04 (0.97-1.11) | **1.33 (1.22-1.44)**  Ref  1.04 (0.96-1.12) |
| **Smoking**   - Never - Past - Occasionally - Daily | Ref  **1.28 (1.21-1.35)**  1.15 (0.98-1.36)  **1.52 (1.41-1.64)** | Ref  **1.16 (1.09-1.23)**  **1.20 (1.02-1.41)**  **1.45 (1.34-1.57)** | Ref  **1.12 (1.05-1.19)**  **1.22 (1.03-1.45)**  **1.49 (1.37-1.63)** | Ref  **1.14 (1.06-1.24)**  1.16 (0.94-1.44)  **1.38 (1.23-1.53)** |

1. Adjusted for sex, age, education, and socioeconomic group, BMI status, physical activity, smoking and comorbidity (diabetes, heart disease, hypertension, osteoporosis and/or sleep apnea)
